# The Importance of Advanced Imaging by Cardiac Magnetic Resonance Imaging in Diagnosing Congenital Left Ventricular Diverticulum

**DOI:** 10.7759/cureus.40302

**Published:** 2023-06-12

**Authors:** Moayad Z Aljuhani, Hussein A Ahmed, Ali A Almasood, Abdullah A Alsehly

**Affiliations:** 1 Internal Medicine, Specialized Medical Center, Riyadh, SAU; 2 Cardiology, Specialized Medical Center, Riyadh, SAU

**Keywords:** adult congenital cardiac malformation, cardiac magnetic resonance imaging, echocardiography, congenital cardiac malformation, left ventricular diverticulum

## Abstract

The left ventricle diverticulum is a very rare congenital cardiac malformation that is usually present during childhood. However, we are reporting the case of an adult patient with a left ventricular diverticulum who presented with chest pain. This paper also highlights the importance of using advanced imaging modalities such as cardiac magnetic resonance, which was essential for diagnosing our case. Our management was conservative, as the patient's chest pain was very mild and resolved within a few days.

## Introduction

The left ventricular diverticulum is a rare congenital cardiac malformation characterized by an outpouching of the left ventricular wall [1-4]. Although it has been reported before, each case has its own unique clinical presentation, anatomy, and imaging characteristics. Here, we present the case of a 55-year-old man who presented with chest pain and was incidentally discovered to have a left ventricular diverticulum during a diagnostic workup. The diagnosis was confirmed by cardiac magnetic resonance imaging, which played a crucial role in establishing the diagnosis and guiding the management strategy. This case highlights the importance of considering the left ventricular diverticulum as a potential cause of chest pain and the value of advanced imaging modalities in the diagnosis and management of this rare cardiac anomaly.

## Case presentation

**Figure 1:**
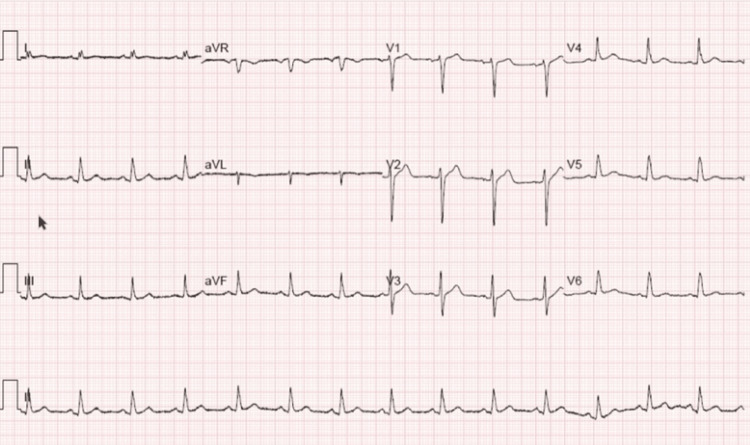
Electrocardiogram Twelve-lead electrocardiogram showing sinus rhythm, without any significant abnormalities.

**Figure 2:**
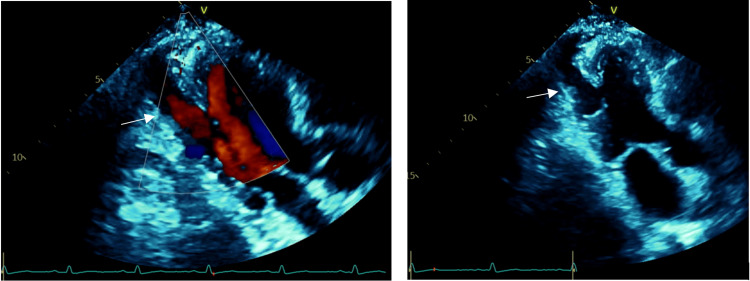
Echocardiogram Echocardiogram of three-chamber view with color compare showing the left ventricular diverticulum - arrow.

A 55-year-old man presented to our cardiology clinic with a sudden onset of sharp left-sided chest pain. The pain has been transient and lasts for a few minutes without any radiation. It is not worsened by exertion, movement, food, activity, or respiration. He also denied shortness of breath, chills, cough, nausea, or diaphoresis. His medical history included diabetes mellitus type 2 and dyslipidemia. He denied any history of myocardial infarction, arrhythmias, hypertension, or stroke. He had never experienced exertional dyspnea or chest pain. His physical examination was unremarkable. An EKG showed a normal sinus rhythm without significant abnormalities (Figure 1). A 2D echocardiogram was done and showed a dissected left ventricular lateral wall with a differential diagnosis of left ventricular diverticulum or pseudoaneurysm. There was normal left ventricular wall thickness. There were no regional wall motion abnormalities (Figure 2).

**Figure 3:**
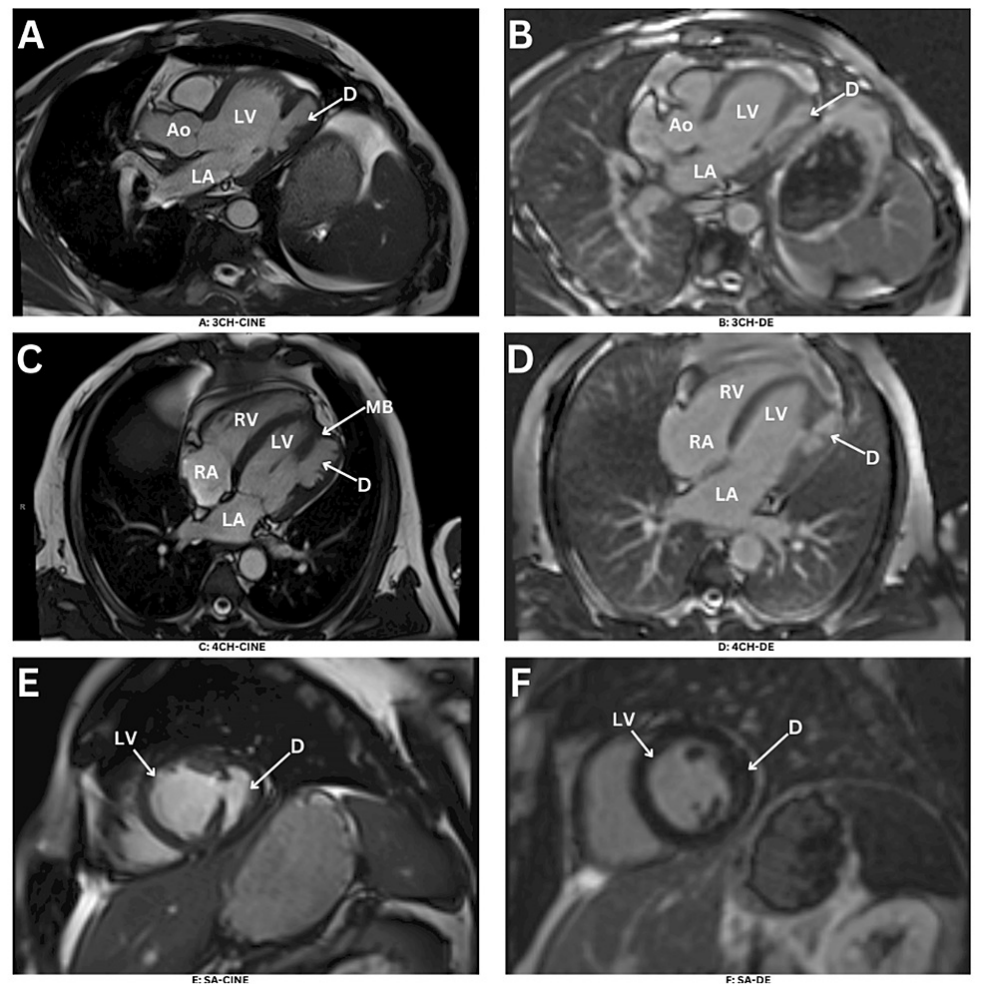
Cardiac magnetic resonance imaging Cardiac MRI demonstrated the left ventricle diverticulum myocardial wall with noticeable wall thinning measuring 2-3 mm in the lateral area in the four chambers long axis SSFP view (C), and D (DE). Three chambers view SSFP sine (A) and (B) of DE. Short axis SSFP (E) and F (DE). D, diverticulum; LA, left atrium; LV, left ventricle; MB, muscle band; RA, right atrium; RV, right ventricle. DE (delayed contrast enhancement). SSFP: steady-state free precession sequence.

Therefore, a cardiac MRI was needed for further assessment. An MRI was done and showed (Figure 3) a large diverticulum of the LV lateral wall. The opening (neck) measures 1.5 cm, and the maximum cavity length was 4.5 cm from a four-chamber view with preserved contractile function and muscular trabeculations/papillary attachment seen inside with mild to moderate hypokinesia seen at the anterior and lateral ventricular walls with noticeable wall thinning in the apical area. Left ventricular ejection fraction (LVEF) 46% cardiovascular magnetic resonance (CMR) tissue characterization sequences and late gadolinium enhancement (LGE) showed no evidence of scarring or fibrosis of the LV or RV myocardium or the diverticulum myocardial wall. No intracardiac thrombi or clots were seen. Left heart catheterization showed no significant lesions.

Our management approach was conservative because the patient's chest pain was mild and resolved within a few days. A six-month follow-up was performed, and the patient remained asymptomatic. The patient will be monitored during his regular clinic appointments to watch for any symptoms or changes that require additional interventions.

## Discussion

The LV diverticulum is a very rare cardiac congenital anomaly in adults. Only 0.4% of cases have been detected at autopsies [1]. The LV diverticulum is a left ventricle outpouching structure that contains all three heart wall layers (endocardium, myocardium, and pericardium) and exhibits synergistic contraction with the left ventricle [2].

Nowadays, the diagnosis of the left ventricular diverticulum relies heavily on advanced imaging. Using cardiac MRI to evaluate cardiac function and visualize the cardiac anatomy has a crucial role in helping to differentiate between similar conditions. Cardiac MRI demonstrates late gadolinium enhancement of the myocardium and pericardium in regions of fibrosis or infarct, which is often seen in cases of left ventricular aneurysm and pseudoaneurysm [3]. On MRI, the LV diverticulum will demonstrate normal myocardial signal intensity in its wall without any discontinuity or enhancement on late-gadolinium sequences. On cine MRI, it will show synchronous contractility [3]. Cardiac magnetic resonance imaging is also well known for its unique and sensitive role in detecting left ventricular thrombi [3]. Earlier studies estimated an incidence of 0.4%, while one study utilizing multi-detector computed tomography angiography found an increased prevalence of 2.2% [3].

There is still debate about whether an isolated, asymptomatic LV diverticulum should be treated with surgery. Typically, surgical therapy is advised when the condition is symptomatic or when it coexists with other cardiac problems [4]. In a few cases, spontaneous regression occurs, and their size may not alter over time, reflecting a benign course. Close clinical monitoring is usually sufficient, and additional management should be dependent on related abnormalities and potential complications [3].

## Conclusions

Congenital left ventricle diverticulum is a rare incidental finding with an unclear appearance on 2D transthoracic echocardiography. This case report stresses the important role of multi-modality imaging in cardiology to establish, categorize, and help in the management strategy of various common and rare congenital and acquired cardiac diseases. Our patient is managed conservatively since no major supporting clinical evidence warrants surgical intervention.
